# P05.39. Clinical experiences of homeopaths participating in a study of the homeopathic treatment of children with attention deficit/hyperactivity disorder

**DOI:** 10.1186/1472-6882-12-S1-P399

**Published:** 2012-06-12

**Authors:** D Brulé, B Landau-Halpern, V Nastase

**Affiliations:** 1 Riverdale Homeopathic Clinic, Toronto, Ontario, Canada

## Purpose

To explore the clinical experiences of homeopaths participating in an open label pilot study of the homeopathic treatment of children with attention deficit/hyperactivity disorder (ADHD). Specific objectives include to: (1) Explore how treating patients within a clinical trial was similar or different from general practice experiences; (2) Reflect on how clinical practices changed as part of the trial; and (3) Identify issues or lessons for future homeopathic clinical researchers and clinicians.

## Methods

A series of in-person interviews were conducted at month 11 of the clinical trial (2/3 completion) with the two study homeopaths. The clinicians were asked a series of open-ended probing questions to explore their experiences participating in the trial and to reflect on issues they felt significant to the study design.

## Results

The clinicians described the study participants as having a greater degree of ADHD pathology, more co-morbidities, and as taking more medication than their daily practice patients. The clinical approach to the study patients deviated from the homeopaths’ normal practice in the following ways: a greater reliance on “water dosing” (giving medication dissolved in water and having the participant dose more frequently) to adapt to concomitant conventional medication; and less dietary advice was given due to the need to establish rapport and the feeling that such advice would overwhelm the families. The homeopaths described challenges in explaining homeopathic treatment concepts such as remedy reaction trajectory and homeopathic remedy aggravations.

## Conclusion

Homeopathic medications and dietary protocols were found to be easily adapted for use in a clinical trial. These observations provide insights for future research in the area of homeopathic treatment (for ADHD in particular and of homeopathy in general) and provide insights for the potential integration of homeopathic practice into conventional settings.

